# Human Milk IgGs Contain Various Combinations of Different Antigen-Binding Sites Resulting in Multiple Variants of their Bispecificity

**DOI:** 10.1371/journal.pone.0042942

**Published:** 2012-08-13

**Authors:** Sergey E. Sedykh, Valentina N. Buneva, Georgy A. Nevinsky

**Affiliations:** Siberian Division of Russian Academy of Sciences, Institute of Chemical Biology and Fundamental Medicine, Novosibirsk, Russia; University of South Florida College of Medicine, United States of America

## Abstract

In the classic paradigm, immunoglobulins represent products of clonal B cell populations, each producing antibodies (Abs) recognizing a single antigen. There is a common belief that IgGs in mammalian biological fluids are monovalent molecules having stable structures and two identical antigen-binding sites. However, human milk IgGs to different antigens undergo extensive half-molecule exchange. In the IgGs pool, only 33±5% and 13±5% of Abs contained light chains exclusively of kappa- or lambda-type, respectively, while 54±10% of the IgGs contained both kappa- and lambda- light chains. All Ab preparations contained different amounts of IgGs of all four subclasses. Interestingly, lambda-IgGs contained an increased amount of IgG2 (87%) and only 3–6% of each of IgG1, IgG3, and IgG4, while kappa-IgGs consisted of comparable (17–32%) amounts of all IgG subtypes. Chimeric kappa-lambda-IgGs consisted of ∼74% IgG1, ∼16% IgG2, ∼5% IgG3 and ∼5% IgG4. As the result of the exchange, all IgG fractions eluted from several specific affinity sorbents under the conditions destroying strong immunocomplexes demonstrated high catalytic activities in hydrolysis of ATP, DNA, oligosaccharides, phosphorylation of proteins, lipids, and oligosaccharides. *In vitro*, an addition of reduced glutathione and milk plasma to two IgG fractions with different affinity for DNA-cellulose led to a transition of 25–60% of Ab of one fraction to the other fraction. Our data are indicative of the possibility of half-molecule exchange between milk IgGs of various subclasses, raised against different antigens (including abzymes), which explains the polyspecificity and cross-reactivity of these IgGs.

## Introduction

There was a common belief that IgGs are monovalent molecules having stable structures and two identical antigen-binding sites [1]–[4]. Recently, it was shown that human IgG4 antibodies are dynamic molecules that exchange Fab arms by swapping a heavy chain and the attached light chain (half-molecule) with a heavy–light chain pair from another molecule, which results in bispecific Abs [1]–[4]. Reduced glutathione (GSH) together with some blood proteins stimulates the exchange *in vitro* leading to formation of hybrid molecules from two different IgG4 [1]–[4]. The formation of bispecific IgG4 was also revealed *in vivo* [1]. However, there is no data concerning a possibility of the exchange between IgGs of different subclasses (IgG1, IgG2, IgG3 and IgG4), lambda-IgGs, and kappa-IgGs.

During the past two decades it has become clear that auto-antibodies (auto-Abs) from the sera of patients with different autoimmune diseases can possess enzymatic activities [5]–[7]. Natural catalytically proficient antibodies (abzymes) hydrolyzing DNA, RNA, polysaccharides, oligopeptides, and proteins are described from the sera of patients with several autoimmune diseases (for a review, see [5]–[7]). Similarly to artificially induced abzymes against analogs of transition states of catalytic reactions [5], naturally occurring abzymes may be Abs raised directly against the enzyme’s substrates acting as haptens and mimicking transition states of catalytic reactions [5]–[9]. On the other hand, antiidiotypic Abs can be induced in autoimmune diseases by a primary antigen and may show some of its features including the catalytic activity [10]–[11].

During pregnancy and immediately after delivery, women are often characterized by immune processes similar to those in autoimmune patients ([6]–[7] and refs therein). Many autoimmune pathologies can be activated or triggered in clinically healthy women during pregnancy and soon after childbirth [12]–[13]. Convincing evidence was provided using different approaches that DNase, RNase [14]–[15], amylase [16], ATPase [17], protein kinase [18], lipid kinase [19], and polysaccharide kinase [20]–[21] activities are intrinsic to human milk Abs. In contrast to canonical kinases, milk IgGs and sIgAs possess a unique capability to phosphorylate their substrates in the presence of [^32^P]*ortho*phosphate [18]–[21].

In this report, we have used several methods to provide the first evidence that molecules of human milk IgGs of different subclasses can contain various combinations of two different antigen-binding sites resulting in multiple bispecificity of milk antibodies and abzymes.

## Results

In this work, homogeneous polyclonal IgG (pIgG) was purified from human milk as in [19]–[21]. Similarly to previously published findings [14]–[21], it was shown that the pIgGs contain subfractions efficiently hydrolyzing DNA, ATP and oligosaccharides. Other subfractions were able to phosphorylate proteins, as well as oligosaccharides and lipids that were tightly bound to the Abs. All these activities were intrinsic to milk abzymes.

**Figure 1:**
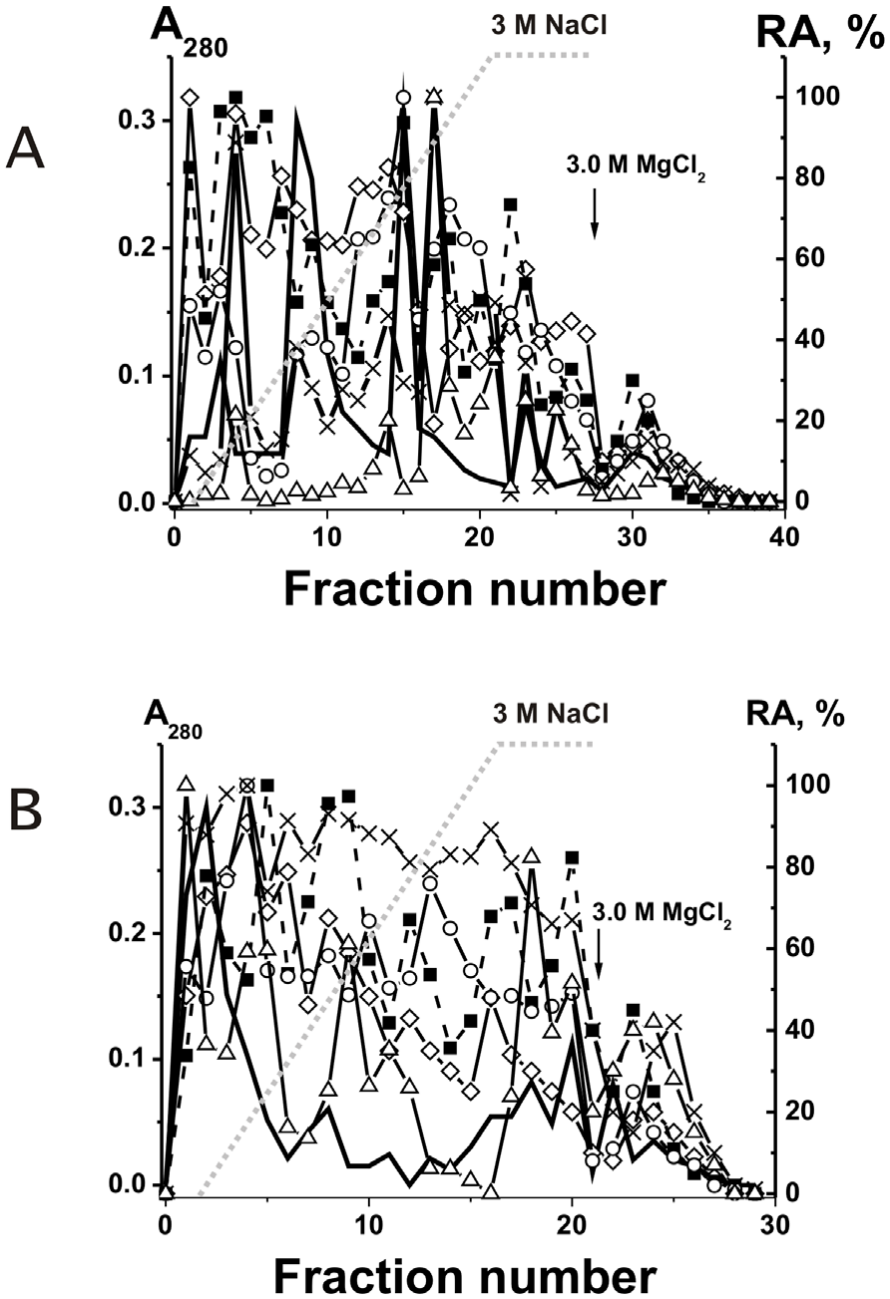
Affinity chromatography of milk pIgGs on two different resins. Various IgG fractions were separated using DNA-cellulose (A) and ATP-Sepharose (B): (–), absorbance at 280 nm; symbols correspond to the relative catalytic activities (RA) in the hydrolysis of DNA (▵), ATP (○), oligosaccharides (×); phosphorylation of lipids (▪) and polysaccharides (⋄) tightly bound with IgGs. Depending on the RA and reaction analyzed, the reaction mixtures were incubated for 0.5–2 h and then the RAs were normalized to the standard conditions and the RA of the fraction with the highest activity was taken for 100%. The average error in the initial rate determination from two experiments in each case did not exceed 7–10%.

Polyclonal IgGs with different catalytic activities are usually very heterogeneous in their affinity for different specific substrates and can be separated into many subfractions by chromatography on specific affinity sorbents [6], [7], [14]–[21]. We have obtained pIgGs from the milk of five women and analyzed their affinity for DNA by chromatography on DNA-cellulose. When individual pIgGs were eluted from DNA-cellulose with a NaCl concentration gradient (0–3 M) and 3 M MgCl_2_, the protein and DNase activity were distributed all over the chromatography profiles. Fig. 1A demonstrates representative data for one of five individual pIgGs. In contrast to previous studies [6]–[7], [15], we have analyzed relative catalytic activities (RAs) of the IgG fractions eluted from DNA-cellulose not only in the hydrolysis of plasmid DNA, but also in the hydrolysis of ATP and oligosaccharides as well as in phosphorylation of proteins, lipids and oligosaccharides (Fig. 1A).

**Figure 2:**
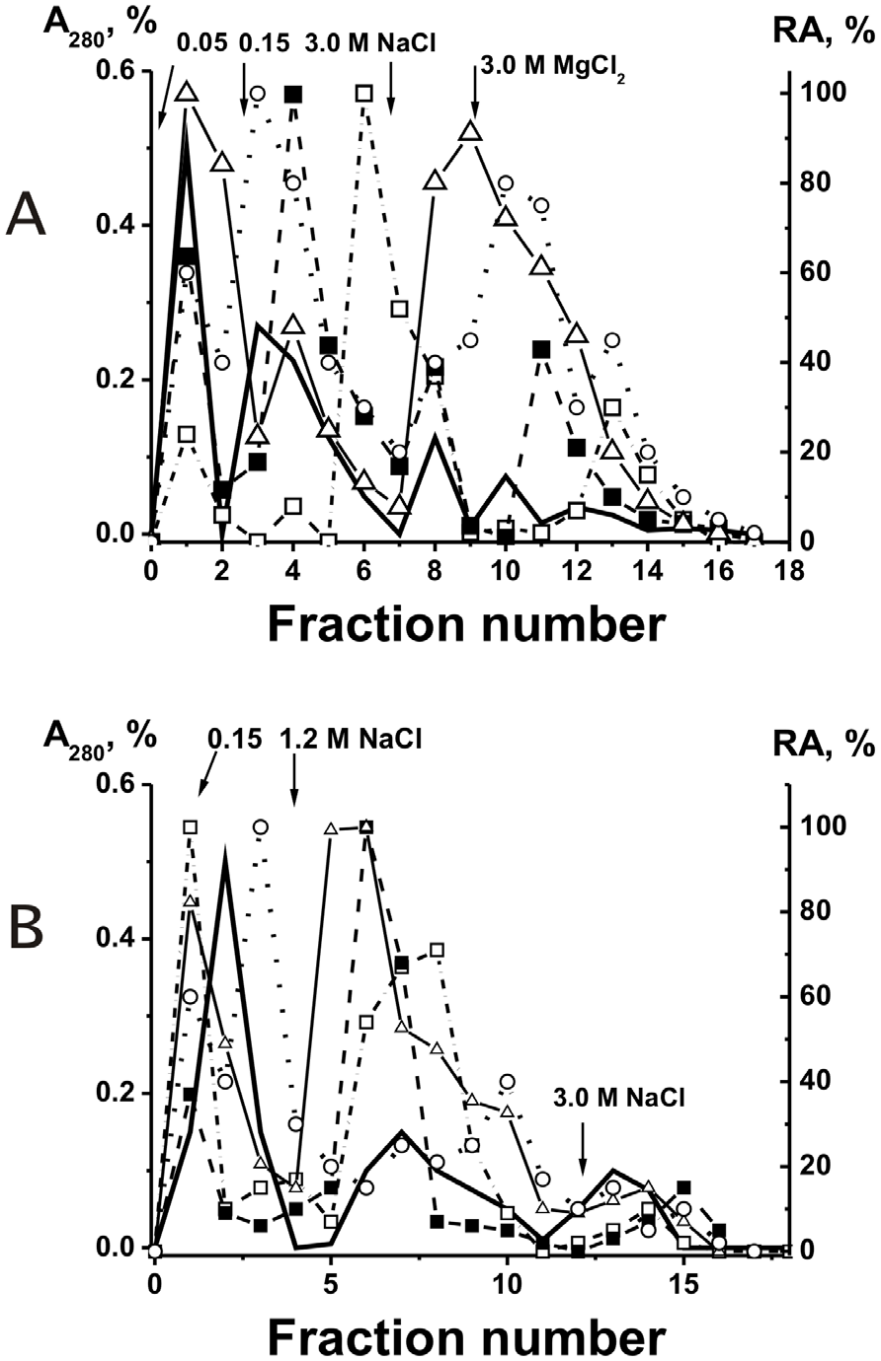
Chromatography of milk pIgGs on two affinity resins. Different IgG fractions were separated using casein-Sepharose (A) and lipid-resin (B): (–), absorbance at 280 nm; symbols correspond to the relative catalytic activities (RA) in the hydrolysis of DNA (▵), ATP (○), casein (□), and phosphorylation of lipids tightly bound with Abs (▪). Depending on the RA and reaction analyzed, the reaction mixtures were incubated for 0.5–3 h and then the RAs were normalized to the standard conditions and the RA of the fraction with the highest activity was taken for 100%. The average error in the initial rate determination from two experiments in each case did not exceed 7–10%.

Polyspecificity is defined as the ability of a given Ab molecule to bind a large panel of structurally diverse antigens. Some studies demonstrated the existence of a large number of monoclonal Abs that can bind to a variety of totally unrelated self and foreign antigens (for review see [22]). Therefore, it was proposed that the best explanation for the polyreactivity is that the antigen-binding ‘pocket’ of many Ab molecules may be flexible and can change conformation to accommodate different antigens [22]. From one side, one cannot exclude that that in the case of some Abs an increase in salt concentration can be associated with a change of Abs conformation leading to their polyspecificity. However, all nonspecific interactions between Abs (or enzymes) and foreign ligands can usually be completely (or at least to a significant extent) destroyed by 0.2–0.5 M NaCl [6]–[7], [22]–[25]. For example, we have previously shown that mouse monoclonal IgGs against ATP can interact with DNA but posses 3–4 orders of magnitude lower affinity to DNA then to Abs against DNA and they can be eluted from ATP-Sepharose by ≤0.05 M NaCl [6], [24]. In addition, canonical enzymes can sometimes interact nonspecifically with foreign ligands demonstrating lower affinity then to specific substrates, but they usually cannot catalyze conversion of molecules of non-cognate compounds [23] (see below). Therefore, it was surprising that all IgG fractions, including those eluted under the conditions destroying strong complexes of Abs with specific antigens (1–3 M NaCl and even 3 M MgCl_2_), not only demonstrated high DNase activity but also efficiently hydrolyzed ATP and oligosaccharides, and phosphorylated casein, oligosaccharides and lipids (Fig. 1A). Therefore, we have estimated the affinity of the same five pIgG preparations for ATP, casein, and lipids by chromatography on ATP-Sepharose, casein-Sepharose, and lipid-saturated silicagel. Again, all enzymatic activities of the Abs were distributed all over the chromatography profiles (Fig. 1 and Fig. 2), suggesting that human milk might contain not only monofunctional abzymes but also hybrid bifunctional IgGs with different combination of HL fragments of H_2_L_2_ IgG molecules, possessing different catalytic activities.

**Figure 3:**
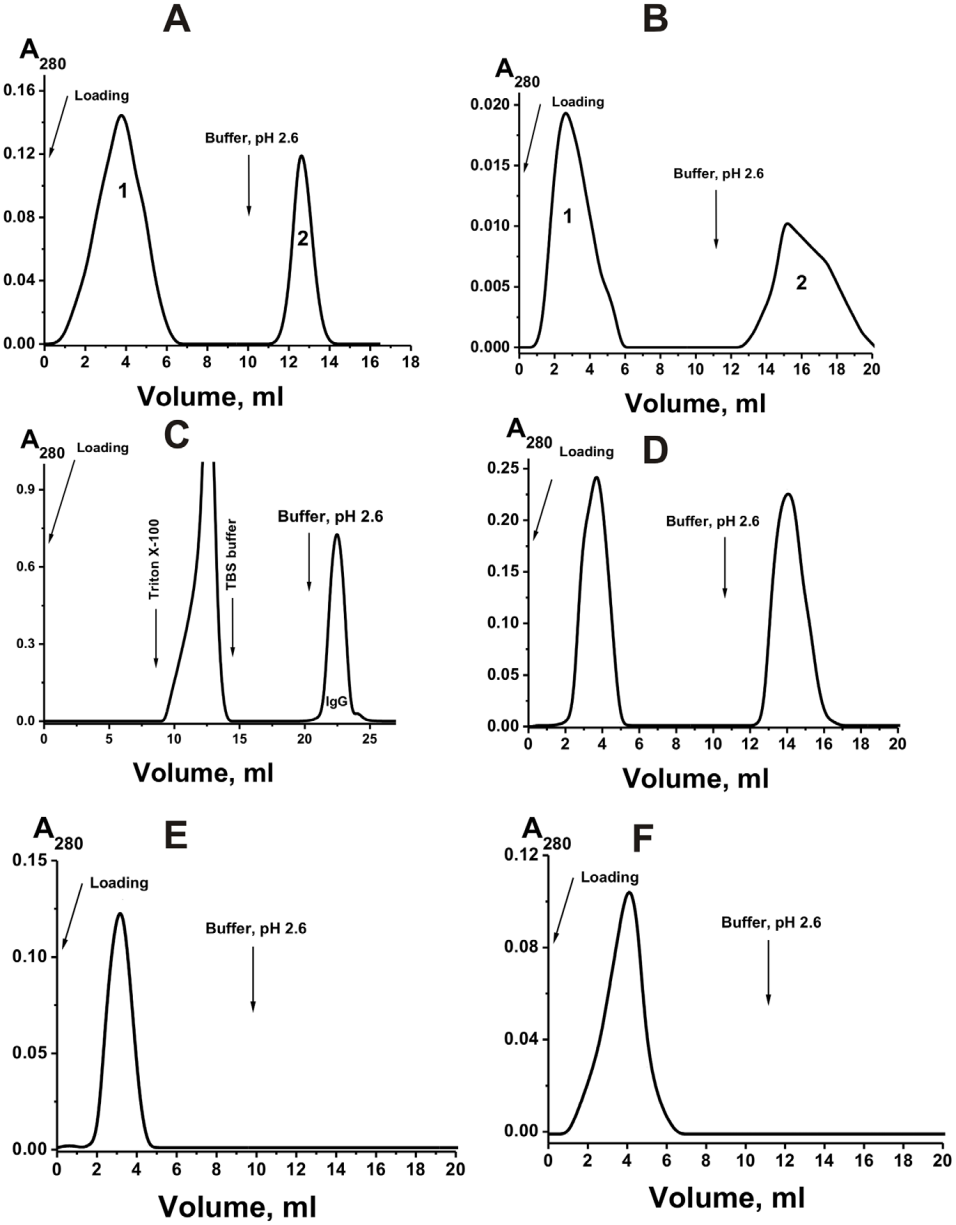
Affinity chromatography of milk pIgGs on anti-kappa-L-Sepharose. The chromatography was performed under the conditions of over-saturation of the affinity capacity of the sorbent (A) and re-chromatography of the preparation eluted by acidic buffer (peak 2) on anti-lambda-L-Sepharose (B) under the conditions of the excess of the affinity sorbent. After incubation of a mixture of equal amounts of purified lambda- and kappa-IgGs for 24 h, lambda+kappa-IgG_mix_ was subjected to standard affinity chromatography on Protein G-Sepharose (C) and gel filtrated (data not shown). Then lambda- and kappa-IgGs were separated by affinity chromatography of lambda+kappa-IgG_mix_ on anti- lambda-L-Sepharose (D). The preparation of lambda-IgGs purified on anti-lambda-L-Sepharose was rechromatographed on anti-kappa-L-Sepharose (E), while kappa-IgGs on anti-lambda-L-Sepharose (F). In all cases: (–), absorbance at 280 nm (A_280_).

Formation of bispecific IgG4 was revealed not only *in vitro*, but also *in vivo* [1]. To reveal a possible chain exchange, milk IgGs were separated by affinity chromatography on Sepharose bearing immobilized monoclonal Abs to human IgGs containing kappa- (anti-kappa-L-Sepharose) and lambda-type (anti-lambda-L-Sepharose) of light chains under the conditions of an excess of the affinity sorbent. It was shown that, depending on the milk donor, anti-kappa-L-Sepharose could bind 60±10%, while anti-lambda-L-Sepharose, 40±8% of the total IgGs. To screen out nonspecific interactions, we then have re-isolated IgG fractions on anti-kappa-L-Sepharose and anti-lambda-L-Sepharose under the conditions of over-saturation of the affinity capacity of the sorbents (Fig. 3A). The IgG fraction having affinity for anti-kappa-L-Sepharose was re-chromatographed on anti-lambda-L-Sepharose (Fig. 3B). Depending on the individual preparation, 45±7% of IgGs having affinity for anti- kappa-L-Sepharose was bound by anti-lambda-L-Sepharose. Conversely, when IgG fraction having affinity for anti-lambda-L-Sepharose was re-chromatographed on anti-kappa-L-Sepharose, only 33±5% of the Abs was eluted in the flow-through, while 67±7% were bound by immobilized anti-kappa-Abs.

The data indicate for a possibility of half-molecule exchange between intact lambda- and kappa-IgGs leading in human milk to a formation of chimeric lambda-kappa-IgGs. However, one could not exclude that the exchange reaction can occur (at least to some extent) during IgG purification or some other manipulation with Ab preparations. Therefore, we have prepared a mixture of equal amounts of purified lambda- and kappa-IgG preparations (lambda+kappa-IgG_mix_ containing no chimeric lambda-kappa-IgGs) which was incubated for 24 h. Then, this mixture was subjected to standard affinity chromatography on Protein G-Sepharose followed by standard gel filtration under the conditions that remove non-specifically bound proteins similarly to purification of IgGs from human milk (Fig. 3C). To reveal a possible chain exchange during purification procedures, the lambda+kappa-IgG_mix_ was separated for lambda- and kappa-IgGs by affinity chromatography on Sepharose bearing immobilized monoclonal Abs to human IgGs containing kappa- and lambda-type of light chains. For example, Fig. 3D shows the data for anti-lambda-L-Sepharose. The preparations of lambda-IgGs purified on anti-lambda-L-Sepharose (and on anti-kappa-L-Sepharose) were rechromatographed on anti-kappa-L-Sepharose (for example, Fig. 3E), while kappa-IgGs on anti-lambda-L-Sepharose (for example, Fig. 3D). In contrast to IgGs purified on anti-lambda-L-Sepharose directly from the samples of milk total IgGs, preparations of lambda-IgGs obtained using mixture of purified lambda- and kappa-IgGs did not contained admixtures of kappa-IgGs (Fig. 3E). Similar result was observed for preparations of kappa-IgGs demonstrating no any admixtures of lambda-IgGs (Fig. 3F). In addition, using ELISA it was confirmed that IgGs purified on anti-lambda-L-Sepharose do not contain kappa-IgGs, while IgGs purified on anti-kappa-L-Sepharose are free of lambda-IgGs. These data show that the exchange reactions do not occur during antibody purification and other standard manipulation with Abs. In addition, the findings demonstrate that specific anti-lambda-L-Sepharose bind only lambda-IgGs while anti-kappa-L-Sepharose only kappa-IgGs. At the same time, both affinity resins interact with chimeric IgGs containing simultaneously lambda- and kappa-chains (Fig. 3B) This result was confirmed by ELISA analysis of purified preparations of lambda-IgGs and kappa-IgGs (see below).

**Figure 4:**
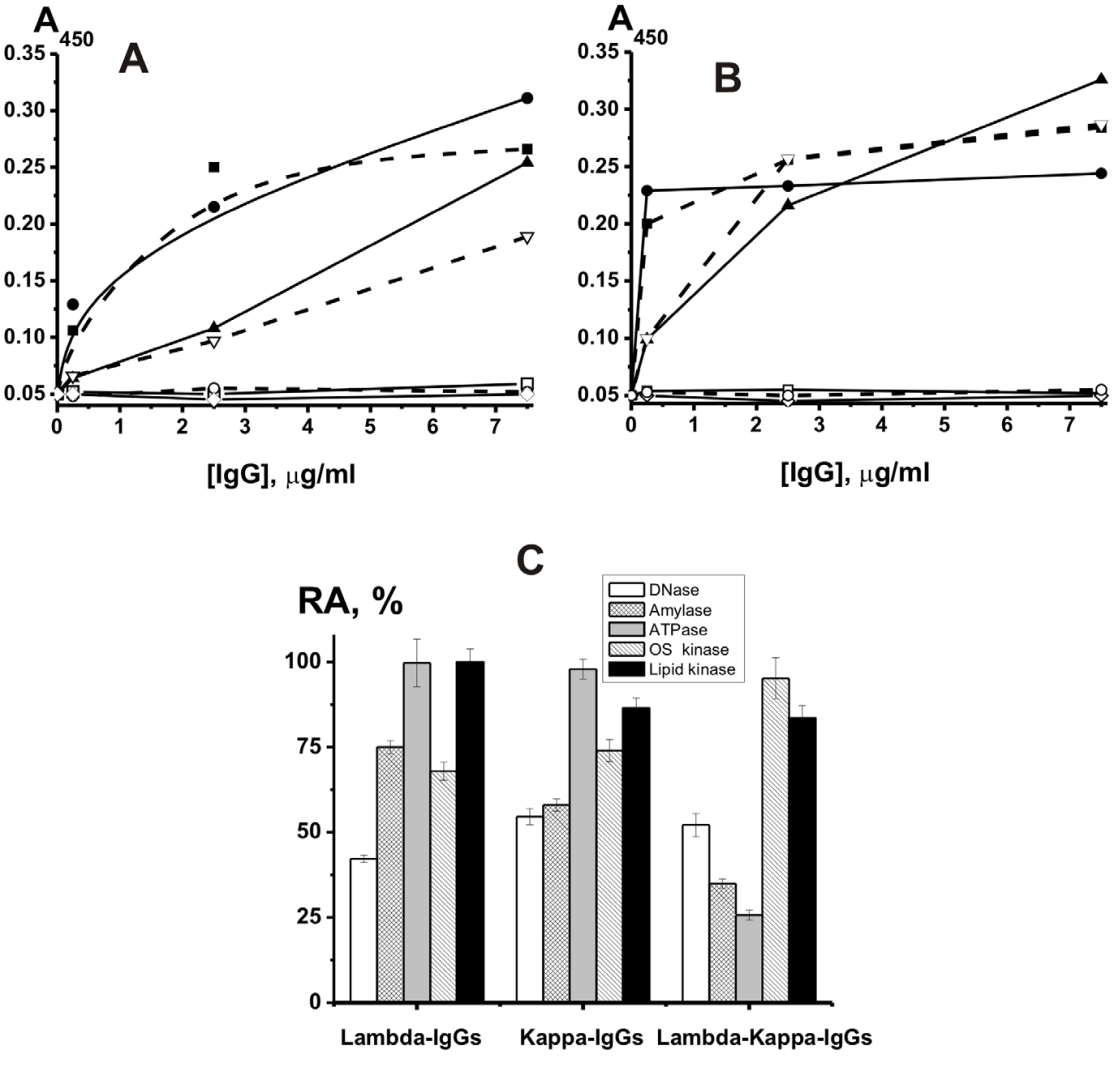
The dependencies of the absorbance (A_450_) on the concentration of tested IgG preparation. The analysis was performed using a direct (A) and sandwich (B) ELISA. For a direct analysis, the tested IgG preparations were adsorbed on ELISA strips, then mouse Abs against human kappa-IgGs or lambda-IgGs (anti-kappa-Abs and anti-lambda-Abs) were added, and finally the conjugate of polyclonal rabbit anti-mouse IgGs with horseradish peroxidase (HRP) was used (C). For a sandwich analysis, anti-kappa-Abs and anti-lambda-Abs were adsorbed on ELISA strips, then tested IgG preparations were added, and finally the conjugate of HRP with mouse Abs against human kappa-IgGs or lambda-IgGs (anti-x-Ab-HRP) were used (B). Anti-lambda-Abs (or anti-lambda-Abs and anti-lambda-Ab-HRP) were used for the analysis of the content of IgGs having affinity only for anti-lambda-Ab-Sepharose (▪), for anti-kappa-Ab-Sepharose (□), and for both of these Sepharoses (▴), while anti-kappa-Abs (or anti-kappa-Abs and anti-kappa-Ab-HDP) were used for the analysis of preparations having affinity only for anti-kappa-Ab-Sepharose (•), for anti-lambda-Ab-Sepharose (○), and for both of these Sepharoses (▾). (⋄), a control mixture containing no analyzed IgGs (A and B). The RAs of lambda-IgGs, kappa-IgGs, and kappa-lambda-IgGs in the catalysis of different chemical reactions (C). The RA of the preparation with the highest activity in each reaction analyzed was taken for 100%. See Materials and Methods for other details.

Using direct (Fig. 4A) and indirect sandwich (Fig. 4B) ELISA, it was shown that only 33±5% and 13±5% of the total pIgGs demonstrated affinity only for light chains of kappa- or lambda-type, respectively, while 54±10% of the IgGs effectively interacted with both anti- lambda-L- and anti-kappa-L-Sepharose. In addition, it was shown that after purification of lambda- and kappa-IgGs from chimeric lambda-kappa-IgGs, these preparations give ELISA positive answer only in the case of cognate anti- lambda- and kappa-Abs, respectively (Fig. 4).

We have shown that the total non-fractionated milk IgGs from five donors contained, on average, 49.7±7.0% IgG1, 30.5±5.0% IgG2, 11.2±3.0% IgG4, and 8.6±2% IgG3 (Table 1). Interestingly, lambda-IgGs contained an increased amount of IgG2 (87%) and only 3–6% each of IgG1, IgG3, and IgG4, while kappa-IgGs consisted of in some extent comparable (17–32%) amounts of all IgG subtypes. The most surprising finding was that preparations of kappa-lambda-IgGs contained mostly IgG1 (∼74%) and significantly lower amounts of IgG2–IgG4 (5–16%) (Table 1), suggesting that IgGs of all four subclasses can participate in the exchange, but IgG1 is the most easily exchangeable.

**Table 1:** The relative content of IgGs of different subclasses in the preparations of total non-fractionated milk IgGs, and in specific lambda-IgGs, kappa-IgGs, and kappa- lambda-IgGs*.

IgG preparation	Relative content, %**			
	IgG1	IgG2	IgG3	IgG4
Total IgGs	49.7±7.0	30.5±5.0	8.6±2	11.2±3.0
lambda-IgGs	6.0±0.6	87.0±8.0*	3.0±0.3	5.0±0.4
kappa-IgGs	27.0±3.0	32.0±2.5	17.0±1.5	24.0±2.0
kappa-lambda-IgGs	74.0±7.0	16.0±1.5	5.0±0.4	5.0±0.5

* Average data for IgGs from five different donors of milk are given.

** For each fraction, a mean of tree repeats is used.

We have analyzed the relative activity of IgGs of a different composition from one milk donor in the catalysis of several reactions (Fig. 4C). The ratio of the RAs corresponding to the different reactions was individual for lambda-IgGs, kappa-IgGs, and kappa-lambda-IgGs.

**Figure 5:**
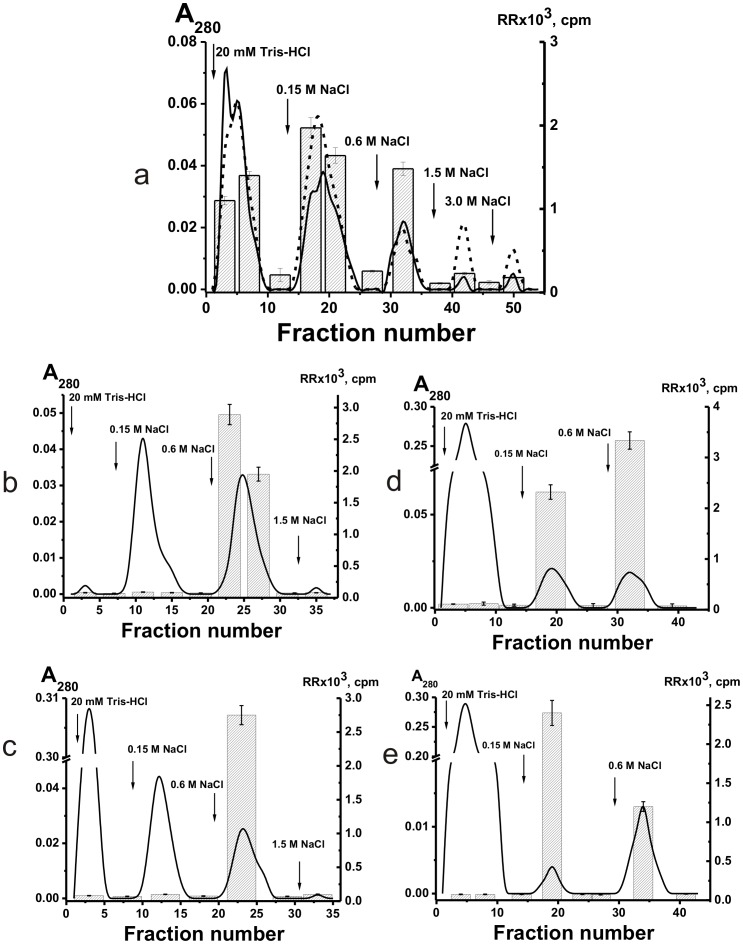
Affinity chromatography of non-modified and ^32^P-labeled pIgGs on DNA-cellulose. (–) and (–), absorbance of IgGs at 280 nm before and after phosphorylation using γ-[^32^P]ATP, respectively; the bars correspond to the relative radioactivity (RR) of [^32^P]IgG fractions (A). Analysis of a relative efficiency of half-molecule exchange under different conditions between non-modified pIgGs and [^32^P]pIgGs having different affinity for DNA-cellulose (B–E). Before chromatography, the IgG preparations eluted from DNA-cellulose by 0.15 M NaCl (0.15 M-IgG_mix_) were incubated with [^32^P]pIgG_mix_ eluted by 0.6 M NaCl (0.6 M-[^32^P]pIgG_mix_) in the presence of TBS and GSH (B) or in the presence of TBS and milk plasma containing no Abs (C); 0.15 M-IgG_mix_ and 0.6 M-[^32^P]pIgG_mix_ (D) or 0.15 M-[^32^P]pIgG_mix_ and 0.6 M-IgG_mix_ (E) were incubated in the presence of TBS containing GSH and human milk plasma.

**Figure 6:**
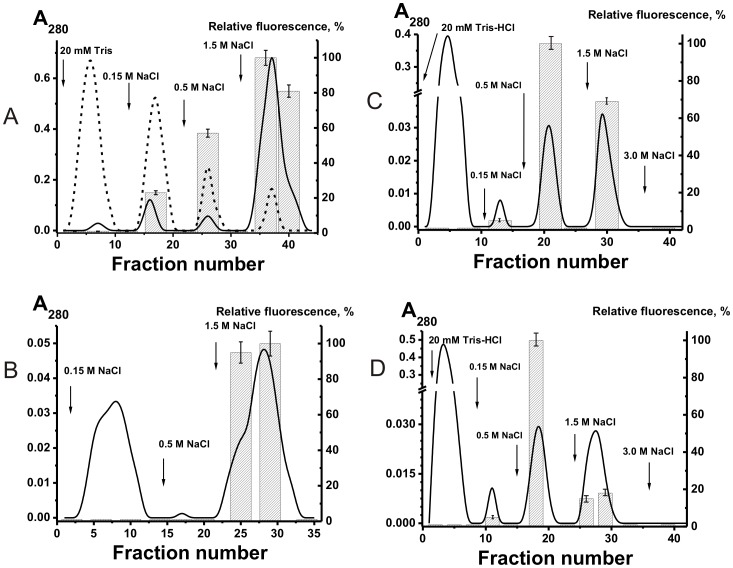
Affinity chromatography of non-modified and FITC-modified pIgG_mix_ on DNA-cellulose. (–) and (–), absorbance of IgGs at 280 nm before and after modification of IgGs with FITC, respectively; the bars correspond to the relative fluorescence of FITC-IgG_mix_ fractions (A). Analysis of a relative efficiency of specific-molecule exchange under different conditions between non-modified IgG_mix_ and FITC-IgG_mix_ having different affinity for DNA-cellulose (B–D). Before chromatography, the IgG_mix_ eluted from DNA-cellulose by 0.15 M NaCl (0.15 M-IgG_mix_) were incubated with FITC-IgG_mix_ eluted by 1.5 M NaCl (1.5 M-FITC-IgG_mix_) in the presence of TBS and GSH (B); 0.5 M-IgG_mix_ and 1.5 M-FITC-IgG_mix_ (C) or 0.5 M-FITC-IgG_mix_ +1.5 M-IgG_mix_ (D) were incubated in the presence of TBS containing GSH and human milk plasma.

To analyze an “average” situation of possible exchange, we have prepared a mixture of equal amounts of pIgGs (pIgG_mix_) from milk of five donors. We have separated the IgG_mix_ before and after its phosphorylation using γ-[^32^P]ATP and protein kinase (or modification by FITC) to five Ab subfractions eluted from DNA cellulose by Tris-buffered saline (TBS; peak 1) or by 0.15–3.0 M NaCl (peaks 2–5) (Fig. 5A). The incubation of pIgG_mix_ eluted from DNA-cellulose with different concentrations of NaCl in TBS or in this buffer containing only reduced glutathione (GSH) or only milk plasma did not lead to an exchange (Figs. 5B and 5C). The situation was changed dramatically after the addition of both GSH and milk plasma to the exchange mixtures. As a result of the exchange, after incubation of 0.15 M-IgG_mix_ and 0.6 M-[^32^P]IgG_mix_ (Fig. 5D) or 0.15 M-[^32^P]IgG_mix_ and 0.6 M-IgG_mix_ (Fig. 5E) in the presence of plasma and GSH, the ^32^P-label was distributed between two peaks: 31±2% and 41±3% of the total ^32^P-label was moved to the initially non-radioactive IgG_mix_. Similar results were obtained in the case of IgG_mix_ with labeled with FITC. After the exchange, 25–60% of FITC-IgG_mix_ changed the affinity for DNA-cellulose (Fig. 6).

**Figure 7:**
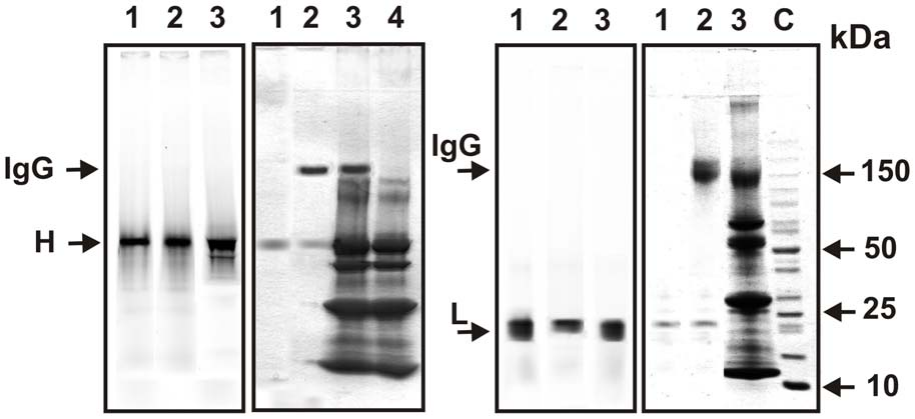
SDS-PAGE analysis of FITC-modified pIgG_mix_ formation. The analysis was performed after non-labeled pIgG_mix_ incubation with preparations of separated FITC-modified heavy (A, B) and light chains (C, D) of Abs. The relative fluorescence of proteins (A and C) and their position on the gel (B and D; Coomassie staining) were analyzed. Before electrophoresis, FITC-heavy-chains were incubated alone (lanes 1, A and B), in the presence of the IgG_mix_ and GSH (lanes 2, A and B) or in the presence of the IgG_mix_, GSH and human milk plasma (lanes 3, A and B). Lane 4 (B) corresponds to human milk plasma incubated alone. FITC-L-chains were incubated alone (lanes 1, C and D), in the presence of the IgG_mix_ and GSH (lanes 2, C and D) or in the presence of the IgG_mix_, GSH, and human milk plasma (lane 3 C and D). The arrows (lane 4, D) indicate the positions of molecular mass markers. For details, see Materials and methods.

In the case of IgG4 a half-molecule (HL-fragments) exchange was recently proposed [1]–[4]. However, there was no data concerning possibility of the IgG4 exchange by only light or heavy chains. Therefore, we have obtained FITC-labeled preparations of light and heavy chains of milk IgG_mix_. Then a possibility of non-modified IgG_mix_ preparation labeling after its incubation with FITC-L- and FITC-H-chain preparations in the absence and in the presence of plasma and GSH was analyzed. It was shown, that in contrast to the exchange of intact IgGs, incubation of IgG_mix_ with separated FITC-modified light and heavy chains does not lead to the exchange; there was not revealed FITC-labeled intact IgGs by SDS-PAGE (Fig. 7).

## Discussion

The ability of some Ab molecules to bind a large panel of structurally diverse antigens is known as polyspecificity or polyreactivity of Abs. It is common to believe that the antigen-binding ‘pocket’ of many Ab molecules may be flexible and can change conformation to accommodate different antigens which leads to Ab polyreactivity. However, affinity of polyreactive Abs for the specific antigens is usually several orders of magnitude higher than their affinity for the non-specific antigens [24], [25]. Some canonical enzymes can also interact with nonspecific ligands [23]. However, the affinity of such enzymes for their specific substrates is usually at least 1–3 orders of magnitude higher than for the nonspecific ligands [23], [26]. It is widely believed that all enzyme-dependent changes in the substrate conformation are necessary for a very precise alignment of electron orbitals of the reacting atoms; it can be achieved only for specific substrates [23], [26]. Therefore, for many enzymes, the conformational adjustment step of the reaction, in contrast to less specific binding, is extremely sensitive to specific elements of the substrate, and it is the catalytic step that determines the reaction rates for different substrates [23], [26]. In contrast to binding, the *k*_cat_ increases by 5–8 orders of magnitude upon a transition from nonspecific ligands to specific substrates [23], [26]. Overall, non-specific binding occurs ubiquitously, while non-specific catalysis is extremely rare.

It was reasonable to expect that all Abs and abzymes nonspecifically interacting with different affinity sorbents can be eluted from the sorbents by 0.1–0.5 M NaCl [6], [7], [24]. Taking into account these data and the very high specificity of enzymes at the stage of the catalysis [6], [7], [23], [26], it was surprising that human milk abzymes catalyzing several very different chemical reactions possessed very high affinity for all affinity sorbents used (Figs. 1 and 2). Some fractions of these abzymes could be eluted from all sorbents only in the conditions that destroy only very specific immunocomplexes. These data could not be explained within the present framework of understanding of Ab polyspecificity. The presence of bifunctional IgG molecules with different types of HL-fragments in human milk can explain the results of the separation of milk pIgG on different affinity sorbents (Figs. 1 and 2).

A direct way to distinguish IgG molecules containing two different HL-fragments is to analyze the possibility of half-molecule exchange between kappa-IgGs and lambda-IgGs. It was shown that only 33±5% and 13±5% of the total pIgGs demonstrated a non-overlapping affinity for light chains of kappa- or lambda-type, respectively, while 54±10% of the IgGs effectively interacted with both anti-lambda-L-Sepharose and anti-kappa-L-Sepharose. Therefore, a formation of bifunctional IgGs is possible in human milk as a result of a specific type of exchange between kappa-kappa-, lambda-lambda-, and kappa-lambda-IgG molecules.

The exchange was demonstrated so far only between two molecules of IgG4, but not between IgGs of other subclasses [1]–[4]. It was proposed that IgG4 molecules can exchange by half- HL-fragments of the antibodies [1]–[4].

We have not observed any IgG exchange in TBS buffer from which either GSH or human plasma was omitted (Fig. 5). An extensive exchange, 25–60%, was found only in the presence of GSH together with human plasma (Figs. 5 and 6).

We have analyzed whether IgG molecules can exchange by only light or heavy chains. After standard incubation of IgG_mix_ with isolated FITC-labeled light and heavy chains in the condition of an effective exchange in the case of intact labeled and non-modified IgGs, FITC-labeled IgGs was not observed (Fig. 7). Thus, our data are indicative of the possibility of only half-molecule exchange between milk IgGs of various subclasses, raised against different antigens and possessing catalytic activities.

Interestingly, the content of IgG1, IgG2, IgG3, and IgG4 was different in our preparations of kappa-IgGs, lambda-IgGs and kappa-lambda-IgGs (Table 1). In addition, the molecules of kappa- lambda-IgGs consisted mainly of HL-fragments corresponding to IgG1 (74%) and lower amounts of HL-fragments of IgG2 (16%), and especially IgG3 and IgG4 (5%). This means that IgG4 and IgG3 cannot be considered as the main type of IgG molecules participating in the exchange in human milk. Since the relative content of HL-fragments corresponding to IgG1–IgG4 in the kappa-lambda-IgG preparations decreased in the same order as the relative content of these IgGs in the non-fractionated milk, it cannot be excluded that accidental exchange corresponding to the relative concentrations of IgGs of different subclasses may occur to some extent.

Since the total pool of milk IgGs contained only 11.2% IgG4, it is obvious that IgGs of other subclasses can also participate in the exchange. Since the extensive exchange occurs only in the presence of GSH together with milk plasma, it is reasonable to suggest that some milk components, most probably protein(s) and/or enzyme(s) (similarly to disulphide isomerase and/or FcRn in the case of IgG4 [1]) can stimulate the exchange.

One can speculate that a similar half-molecule exchange can proceed to some extent in the blood of all healthy humans or at least lactating women. This phenomenon should lead to an increase in polyspecificity of polyclonal Abs and to cross-catalytic activity observed by us for the first time for IgGs.

## Materials and Methods

### Chemicals and Donors

Most of the reagents including monoclonal mouse Abs (anti-IgG1, anti-IgG2, anti-IgG3, anti-IgG4, anti-kappa-IgG, or anti-lambda-IgG antibodies) used in this work were obtained from Sigma. We also used Protein G-Sepharose, Protein A-Sepharose from GE Healthcare. Glutarate-crosslinked DNA-cellulose was from NIKTI BAV (Russia). Sepharoses bearing the monoclonal Abs, β-casein, or γ-(aminohexamethylenamide)-ATP (ATP-Sepharose) were prepared using BrCN-activated Sepharose according to the standard manufacturer’s protocol. Samples of milk were taken by obstetricians from five healthy human mothers (19–35-years old) within the period of 1*–*3 weeks after the beginning of lactation during standing in maternity hospital. The milk sampling protocol conformed to the local human ethics committee guidelines (Ethics committee of Novosibirsk Statement Medical University, Novosibirsk, Russia) including written consent of women recently confined to present of an excess of their milk for scientific purposes. According to standard procedure in Russian hospitals all pregnant women before the admission to the maternity hospital should be analyzed for different possible diseases in accordance with Helsinki ethics committee guidelines. Obstetrician-gynecologists gave us milk samples from donors having a negative history of autoimmune, rheumatologic, respiratory, cardiovascular, gastrointestinal, reproductive, or nervous system pathology.

### Purification and Analysis of Antibodies

Five electrophoretically and immunologically homogeneous IgG preparations were obtained by affinity chromatography of breast milk protein on Protein G-Sepharose followed by gel filtration on a Superdex 200 HR 10/30 column under the conditions that remove non-specifically bound proteins similarly to [18], [19]. The SDS-PAGE analysis of the Ab fractions for homogeneity under nonreducing conditions was performed in a 4–16% gradient gel (0.1% SDS); an analysis for the polypeptide spectrum was performed in a reducing 12.5% gel (in the presence of 0.1% SDS and 10 mM DTT) in the Laemmli system as described previously [18], [19]. The gel was silver-stained according to a standard procedure. The type of Abs (sIgA, IgG or IgM) in the fractions was determined by Western blotting on a nitrocellulose membrane as described previously [17]. In order to protect the IgG preparations from bacterial contamination, they were filtered through a Millex filter (pore size 0.2 µm). After 1 week of storage at 4°C for refolding after the “acidic shock”, a necessary step in the purification, the IgGs were used in the activity assays as described below. To exclude possible artifacts due to traces of contaminating enzymes, the IgG activities in the hydrolysis of DNA, ATP, oligosaccharides, phosphorylation of casein, lipids and oligosaccharides were analyzed after SDS-PAGE of IgGs as in [14]–[21]. All activities were revealed only in the band corresponding to intact IgGs and there were no other peaks of proteins or enzymatic activities.

### DNase Activity Assay

DNase activity was analyzed using supercoiled pBluescript plasmid DNA as described earlier [14], [15]. The reaction mixture (20 µl) contained 50 mM Tris-HCl (pH 7.5), 5 mM MgCl_2_, 20 µg/ml DNA, 1 mM EDTA, and 5–30 µg/ml IgGs, and was incubated for 0.5–3 h (standard time, 2 h) at 37°C. The cleavage products were analyzed by electrophoresis in a 1% agarose gel. The images of ethidium bromide-stained gels were captured on a Sony DSC-F717 camera and a relative amount of DNA in different bands was analyzed using ImageQuant v5.2 (Molecular Dynamics). The activities of IgGs were determined as a decrease in the percentage of DNA converted from the initial supercoiled form to the relaxed form (and sometimes additionally linear form), corrected for the distribution of DNA between these bands in the control (incubation of pBluescript in the absence of Abs). All measurements (initial rates) were taken within the linear regions of the time courses and Ab concentration curves.

### ATP-hydrolyzing Activity Assay

ATPase activity was analyzed as in [17]. The reaction mixtures (20 µl) containing 50 mM Tris-HCl (pH 7.5), 1 mM MgCl_2_, 0.3 mM EDTA, 0.2 mM γ-[^32^P]ATP (1×10^7^ cpm) and 20–80 µg/ml IgGs were incubated for 1–3 h at 37°C (standard time, 2 h). The products of ATP hydrolysis were analyzed by thin-layer chromatography on PEI-cellulose plates (Merck) in 0.25 M potassium phosphate (pH 7.0). After the chromatography, the plates were dried and various ^32^P-labeled products were quantified by phosphorimaging (Molecular Imager FX system, Bio-Rad Laboratories, Hercules, CA). The relative amounts of radioactivity in the products were calculated using the scanning data of the spots corresponding to [^32^P]*ortho*phosphate and non-hydrolyzed γ-[^32^P]ATP. The activities of IgGs were determined from percentages of the product in the spots of [^32^P]*ortho*phosphate and non-hydrolyzed γ-[^32^P]ATP. All measurements were taken within the linear regions of the time courses and Ab concentration dependences.

### Amylase Activity Assay

Amylolytic activity was analysed as in [16]. The reaction mixture (15–20 µl) containing 30 mM Tris-HCl (pH 7.5), 1 mM NaN_3_, 1.5 mM maltoheptaose, and 20–80 µg/ml of IgGs was incubated for 6–12 h at 30°C. Products of hydrolysis were identified by TLC on Kieselgel plates (Merck) using 1-butanol–acetic acid–H_2_O (12∶4:4). The activities of IgGs were determined from the scanning data from percentages of oligosaccharides in the spots of maltoheptaose and its hydrolyzed forms. All measurements were taken within the linear regions of the time courses and Ab concentration dependences.

### Protein Kinase Activity Assay

Protein kinase activity of milk IgGs was measured as in [18]. The reaction mixtures (20 µl) contained 50 mM Tris-HCl (pH 6.8), 3 mM MgCl_2_, 0.3 mM EDTA, 50 mM NaCl, 0.6 mg/ml casein, 0.1 mM γ[^32^P]ATP (5×10^7^ cpm), and 50–200 µg/ml IgGs. The product of casein phosphorylation was analyzed by Laemmli SDS-PAGE in a 12.5% gel with Coomassie R250 staining. The relative amount of [^32^P]casein was quantified by phosphorimaging.

### Assay of Lipid and Oligosaccharide Kinase Activities of IgGs

Phosphorylation of lipids and oligosaccharides tightly bound to the IgGs was analyzed as in [19]–[21]. The reaction mixtures (20 µl) contained 10 mM Tris-HCl (pH 7.5), 1 mM MgCl_2_, 0.1 mM EDTA, 70 mM NaCl, 0.1 µM [^32^P]*ortho*phosphate (20 µCi), and 20–100 µg/ml pIgGs. The samples were incubated at 37°C for 2 h. The reaction was stopped by addition of an equal volume of 20% trichloroacetic acid (20 µl), and [^32^P]lipids was extracted with a chloroform–methanol mixture (2∶1). The extracts were evaporated to dryness, the lipids were solved in 10 µl of a chloroform–methanol mixture (1∶1) and analyzed by TLC on Kieselgel 60 plates using the solvent system A, chloroform–methanol–H_2_O (14∶6:1) [19]–[21]. The aqueous phase of the extracted solution containing [^32^P]oligosaccharides was dried, the precipitate was solved in 5–10 µl of water and used for the analysis of [^32^P]OS by TLC on Kieselgel 60 plates using the solvent system B, dioxane–7 M NH_4_OH–H_2_O (5∶1:4) [19]–[21]. The relative amounts of [^32^P]lipids and [^32^P]oligosaccharides were quantified by phosphorimaging.

### Chromatography of IgGs on DNA-cellulose

Electrophoretically homogeneous IgGs were loaded onto a DNA cellulose column (30 ml) equilibrated with 20 mM Tris-HCl (pH 7.5), and the column was washed with the same buffer to zero optical density. The IgGs were eluted using the same buffer and either a gradient of NaCl concentration (0–3 M) or different concentrations of NaCl (0.02, 0.15, 0.3, 1.5, 3.0 M), and then with 3 M MgCl_2_, as in [14], [15]. IgG fractions were collected, dialyzed against 20 mM Tris-HCl (pH 7.5) containing 0.1 M NaCl, concentrated, and each fraction was used in the analysis of various enzymatic activities.

### Chromatography of IgGs on ATP-Sepharose

Purified IgGs were applied to an ATP-Sepharose column (3 ml) equilibrated with 25 mM Tris-HCl (pH 7.5) containing 1 mM MgCl_2_ as in [18]. Unbound proteins were eluted with the same buffer. Adsorbed IgGs were eluted with a gradient of NaCl concentration (0–3 M) in 25 mM Tris-HCl (pH 7.5), and then with 3 M MgCl_2_. Individual fractions were collected, dialyzed against 20 mM Tris-HCl (pH 7.5), concentrated, and their catalytic activities were measured as described above.

### Chromatography of IgGs on Casein-Sepharose

Purified IgGs was subjected to a chromatography on a casein-Sepharose column (7 ml) equilibrated in 20 mM Tris-HCl (pH 7.5) similarly to [27]. After IgG loading, the column was washed with this buffer to zero optical density in the eluate. The bound IgGs were eluted with with the same buffer containing different concentrations of NaCl (50, 150, and 3 M), and then with 3 M MgCl_2_. IgGs were collected, dialyzed against 10 mM Tris-HCl (pH 7.5) containing 0.1 M NaCl, concentrated, and each fraction was used in the assay of various catalytic activities.

### Chromatography of IgGs on Lipid-saturated Silicagel

The sorbent was prepared by saturation of silicagel with a chloroform-methanol (1∶1) extract of the human milk lipid and fat fraction. Purified IgGs were then applied to the column (10 ml) equilibrated in 20 mM Tris-HCl (pH 7.5). Unbound proteins were eluted with the same buffer. The bound IgGs were eluted with with the same buffer containing different concentrations of NaCl (0.05, 0.15, and 1.2 M). IgG fractions were collected, dialyzed against 10 mM Tris-HCl (pH 7.5), concentrated, and each fraction was used in the assay of various catalytic activities.

### Purification of Lambda-IgGs, Kappa-IgGs, and Lambda-kappa-IgGs

IgGs (0.1–1.0 mg) were chromatographed on Sepharose bearing immobilized monoclonal mouse specific Abs to human kappa-IgG or lambda-IgG. The column (1 ml) was equilibrated with 50 mM Tris-HCl (pH 7.5) containing 50 mM NaCl; the protein was applied and then the column was washed with a buffer containing 0.5 M NaCl to zero optical density. IgGs were eluted from the sorbent with 0.1 M glycine-HCl (pH 2.6). The column fractions were collected into cooled tubes containing 50 µl of 0.5 M Tris-HCl (pH 9.0), and were additionally neutralized with this buffer. The fractions having affinity for anti-kappa-Abs were re-chromatographed on anti- lambda-IgG-Sepharose, while IgGs eluted from anti-lambda-IgG-Sepharose, on anti-kappa-IgG-Sepharose. The final fractions were dialyzed against 50 mM Tris-HCl (pH 7.5) containing 50 mM NaCl and concentrated. In order to protect Ab preparations from bacterial contamination, all fractions used were filtered through a Millex syringe-driven filter units (0.2 µm) and kept in sterilized tubes. The preparations obtained were used for ELISA and determination of their catalytic activities.

### Analysis of Effect of IgG Purification Conditions on the Exchange Reaction

Re-purification of the mixture of lambda- and kappa-IgGs was performed similarly to purification of IgGs from human milk (see above). A mixture (1 ml) of equal amounts of purified lambda- and kappa-IgGs without admixture of chimeric lambda-kappa-IgGs containing 150 mM NaCl, 50 mM Tris-HCl, pH 7.5 and 2.2 mg/ml lambda+kappa-IgG_mix_ was incubated at 25°C for 24 h. Then, it was diluted 5 times with buffer A (150 mM NaCl, 50 mM Tris-HCl, pH 7.5) and loaded on a 3-ml protein G-Sepharose column equilibrated in buffer A. The column was washed with 8 ml of buffer A and then with this buffer (5 ml) containing 1% Triton X-100 and 0.3 M NaCl and the column was washed with buffer A to zero optical density. The total IgG_mix_ fraction was eluted in 0.1 M glycine-HCl (pH 2.6), the column fractions were collected to cooled tubes containing 50 µl of 0.5 M Tris-HCl (pH 9.0) and concentrated for the following step of purification. FPLC gel filtration of this fraction was performed on a Superdex 200 HR 10/30 column as in [18]–[19]. The column fractions were collected, neutralized, and dialyzed as described above for IgG purification from human milk; lambda+kappa-IgG_mix_ was used for an analysis of the content of lambda-, kappa-, and lambda-kappa-IgGs.

### ELISA of Different Antibodies

After chromatography of IgGs on Sepharose bearing immobilized monoclonal mouse anti-kappa-IgG or anti-lambda-IgG Abs, the IgGs were analyzed for the content of kappa-IgGs, lambda-IgGs, and IgG1–IgG4 by direct or double sandwich ELISA. For direct ELISA, sodium carbonate (50 µl, pH 9.6) containing 0.4–12 µg/ml of one of the tested IgGs was added to the ELISA strips and incubated overnight at 22°C. The assembled strips were washed once with TBS buffer containing 0.01% NaN_3_ and 0.05% Triton X-100 and then twice with the same buffer without Triton X-100. The strips were blocked for 2 h at 37°C using TBS containing 3% bovine albumin and 0.01% NaN_3_, and washed 10 times with water and then with TBS containing 0.01% NaN_3_.

Each of the monoclonal mouse Abs (100 µl, 0.01 mg/ml; anti-IgG1, anti-IgG2, anti-IgG3, anti-IgG4, anti-kappa-IgG, or anti- lambda-IgG) in TBS containing 3.0% bovine albumin, 0.01% NaN_3_ and 0.05% Triton X-100 was added to the strips corresponding to human IgGs of different subclasses and incubated for 2 h at 37°C. After washing the strips with water (10 times) and TBS, 100 µl TBS containing 1.0% bovine albumin and 0.01% NaN_3_ were added and incubated for 2 h at 37°C. The strips were washed 10 times with water, incubated with 100 µl TBS containing 1 µg/ml conjugate of polyclonal rabbit anti-mouse IgGs with horseradish peroxidase for 30 min at 37°C, and washed again 10 times with water. After an addition of 50 µl of citrate/phosphate buffer containing 3,3′,5,5′-tetramethylbenzidine and H_2_O_2_, the strips were incubated for 15 min at room temperature, and the reaction was stopped by addition of 100 µl of 1 M H_2_SO_4_.

For a double sandwich analysis, monoclonal mouse Abs (0.01 mg/ml; anti-kappa-IgG, or anti-lambda-IgG, Sigma) in 50 µl of sodium carbonate were incubated in ELISA strips for 1 h at 37°C. The assembled strips were washed with TBS and blocked for 2 h at 37°C using TBS containing 3% bovine albumin as described above. After a standard strip wash, one of the tested IgG preparations (50 µl; 5–12 µg/ml) in TBS containing 1% bovine albumin was added and incubated for 2 h at 37°C. After a standard strip wash with water, PBS containing 0.1% Triton X-100, and then PBS, 25 µl of TBS containing a conjugate of horseradish peroxidase with Abs against lambda- or kappa-light chains of human Abs (diluted 7,000–30,000-fold according to a standard manufacturer’s protocol) were added, and the mixtures were incubated at 37°C. After a standard wash, 50 µl of citrate/phosphate buffer containing 3,3′,5,5′-tetramethylbenzidine and H_2_O_2_ were added, the strips were incubated for 15 min at room temperature, and the reaction was stopped by addition of 100 µl of 1 M H_2_SO_4_.

In both direct and double sandwich ELISA, the relative concentrations of analyzed Abs in the samples was expressed as the difference in the relative absorbance at 450 nm (average of three measurements) between the experimental and control data.

### Preparation of Milk Plasma

Milk (10 ml) from healthy human mothers was centrifuged for 1 h at 6000×*g*. The lipid and cell phases were removed, the solution was dialyzed against TBS (150 mM NaCl, 20 mM Tris-HCl pH 7.4), and then filtered twice through Sephadex G-75 (10 ml) to remove fats that have remained in the solution. Then, all Abs were removed from the milk plasma using a sequential affinity chromatography of plasma proteins on protein G-Sepharose (5 ml) and protein A-Sepharose (5 ml) equilibrated with TBS. The flow-through fraction from the affinity sorbents data did not contain Abs according to ELISA.

### Preparation of Labeled IgGs

For ^32^P-labeling of human milk IgGs, the reaction mixture (1 ml) containing 30 mM Tris-HCl (pH 7.5), 10 mM MgCl_2_, 100 nM 3′,5′-cAMP, 10 nM γ-[^32^P]ATP, 20 µg/ml cAMP-dependent protein kinase and 1.5 mg/ml IgGs was incubated for 12 h at 30°C. [^32^P]IgGs were separated from the protein kinase, γ-[^32^P]ATP, and products of its hydrolysis by ultrafiltration using Centricon-100 devices. The product of IgG phosphorylation was analyzed by Laemmli SDS-PAGE in 12.5% gels with Coomassie R250 staining. The relative amount of [^32^P]IgGs was quantified by phosphorimaging.

To obtain IgG preparations modified with fluorescein isothiocyanate (FITC), the reaction mixture (1 ml) containing 0.1 M NaHCO_3_ (pH 8.3), 0.1 mg/ml FITC, and 1–2 mg/ml IgGs was incubated for 36 h at 24°C in darkness. FITC-IgGs were purified by gel filtration on Sephadex G-25 Superfine (1×10 cm) equilibrated with 30 mM Tris-HCl (pH 7.5). The fluorescence of FITC-IgGs was analyzed using a PharosFX imaging system (Bio-Rad; fluorophores mode, FITC, high sample intensity). After concentration, the FITC-IgG preparations were used for the analysis of IgG exchange.

### IgG Exchange Analysis

The reaction mixtures (0.2–0.4 ml) contained 20 mM Tris-HCl (pH 7.5), 0.15 M NaCl, 10–100 mM GSH, equal amounts of labeled and non-labeled IgGs (0.1–0.2 mg/ml) possessing different affinity for DNA-cellulose, and human plasma containing no Abs (1/10 of the total volume). The mixtures were incubated for 48–72 h at 37°C (in darkness in the case of FITC-IgGs) and then dialyzed against 20 mM Tris-HCl. To reduce the disulfide bonds of IgGs, oxidized glutathione was added to 100 mM final concentration, and the reaction mixture was incubated for 24 h at 37°C. Then the reaction mixtures were loaded onto a DNA-cellulose column (8 ml) equilibrated with 20 mM Tris-HCl (pH 7.5), and the column was washed with the same buffer to zero optical density. The IgGs were eluted using the same buffer containing NaCl (0.15, 0.3, 1.5, and 3.0 M). IgG fractions were collected, dialyzed against 20 mM Tris-HCl (pH 7.5), and their relative content of [^32^P]-label or relative fluorescence was determined.

For the reduction of Ab disulfide bonds the reaction mixture (1 ml) containing 0.2 M acetic acid, 0.1 M DTT, 8 M urea, and 2 mg/ml of IgG_mix_ was incubated for 12 h at 37°C as in [28]. Fractionation of reduced light and heavy chain of IgG_mix_ was performed using ion-exchange chromatography on GE HiTrap HP SP column (1 ml) according to [28]. The preparations of light and heavy chains were eluted by different concentrations of NaCl and immediately desalted using GE HiTrap Desalting columns equilibrated with 20 mM sodium carbonate buffer (pH 9) and then these preparations were dialyzed against this buffer and finally modified with FITC using standard procedure (see above). Using SDS-PAGE it was shown that in contrast to the samples of heavy chains, the preparations of free light chains were unstable under conditions of the modification. Therefore, for a obtaining of labeled light chains we first modified IgGs by FITC and after Ab reduction the labeled light chains were separated from other different components of IgGs using Amicon-50. To analyze the exchange 1 mg/ml IgG_mix_ were incubated in the presence of 0.2 mg/ml FITC-L-chain or 0.2 mg/ml FITC-H-chain preparations for 72 h using different conditions described above. Then a formation of FITC-labeled IgGs was analyzed by SDS-PAGE.
